# Impact of COVID-19 pandemic on the epidemiology of STDs in China: based on the GM (1,1) model

**DOI:** 10.1186/s12879-022-07496-y

**Published:** 2022-06-04

**Authors:** Jingmin Yan, Yanbo Li, Pingyu Zhou

**Affiliations:** 1 Shanghai Skin Disease Clinical College of Anhui Medical University, Shanghai Skin Disease Hospital, Shanghai, 200050 People’s Republic of China; 2 Institute of Molecular Plus, Tianjin University, Tianjin, 300072 People’s Republic of China; 3 Sexually Transmitted Disease Institute, Shanghai Skin Disease Hospital, School of Medicine, Tongji University, Shanghai, 200050 People’s Republic of China

**Keywords:** COVID-19, HIV infection, Syphilis, Gonorrhea, Epidemiology, GM (1,1)

## Abstract

**Background:**

COVID-19 and Sexually Transmitted Diseases (STDs) are two very important diseases. However, relevant researches about how COVID-19 pandemic has impacted on the epidemiological trend of STDs are limited in China. This study aimed to analyze the impact of COVID-19 on STDs in China and proposed relevant recommendations to be used in bettering health.

**Methods:**

The incidence of HIV infection, syphilis and gonorrhea in China from 2008 to 2020 were collected. Grey Model (1,1) were established to predict the incidence of STDs with the incidence data of these three STDs from 2013 to 2018 considering the impact of policies in China, respectively. We then calculated the predictive incidence of each STD in 2019, 2020 and 2021 by the established Model. And we estimated the extent of the impact of COVID-19 on the epidemiological changes of STDs by analyzing the difference between the absolute percentage error (APE) of the predictive incidence and actual rate in 2019 and 2020.

**Results:**

The incidence of HIV infection and syphilis showed a trend of increase from 2008 to 2019 in China, but that for gonorrhea was fluctuant. Of note, the incidence of these three STDs decreased significantly in 2020 compared with that in 2019. The APE of HIV infection, syphilis and gonorrhea in 2020 (20.54%, 15.45% and 60.88%) were about 7 times, 4 times and 2 times of that in 2019 (2.94%, 4.07% and 30.41%). The incidence of HIV infection, syphilis and gonorrhea would be 5.77/100,000, 39.64/100,000 and 13.19/100,000 in 2021 based on our model.

**Conclusions:**

The epidemiological trend of STDs in China was significant influenced by COVID-19 pandemic. It is important to balance the control of COVID-19 and timely management of STDs during the COVID-19 epidemic to prevent or reduce the poor outcome among COVID-19 patients with STDs. New management strategies on STDs, such as leveraging social media, online medical care, rapid self-testing, timely diagnosis and treatment guarantee and balance of medical resources for STDs management should be adapted in the context of the long-term effects of COVID-19.

**Supplementary Information:**

The online version contains supplementary material available at 10.1186/s12879-022-07496-y.

## Background

War, natural disaster, epidemic of infectious diseases and other social events would affect people’s life and health [1]. Known as a catastrophe in global infectious disease history, the outbreak of Coronavirus Disease 2019 (COVID-19) pandemic has affected people’s daily life, medical behavior and many other aspects [2, 3]. Researches have indicated that the COVID-19 pandemic has influenced individual’s sexual health, sexual behavior, and the diagnosis and treatment process of Sexually Transmitted Diseases (STDs), which in turn changed the epidemiological trend of STDs [4–7]. It is reported that except Denmark [8], the incidence or reported cases of STDs in Spain, Greece, Cuba, and the US significantly decreased in 2020 compared with the corresponding period in 2019 [5, 9–11].

The prevalence of COVID-19 pandemic is different from country to country, and each country has its own strategies to prevent and control its transmission [11, 12]. The research in Cuba found that under strict social restrictions, the incidence of syphilis and gonorrhea continued to decline, while following subsequent relaxed social measures, the incidence of syphilis was increased [10]. As a country with a sustained rise in the incidence of STDs in recent years [13, 14], however, relevant researches are limited about the influence of COVID-19 on the STDs in China. HIV infection, syphilis and gonorrhea are national statutory infectious diseases in China. In this study we aim to analyze the extent of the COVID-19 pandemic impact on these three STDs in China by using Grey Model (1,1) [GM (1,1)], and try to explore effective recommendations on STDs management in the period of COVID-19 pandemic and STD patients infect with COVID-19.

Grey prediction in the grey system theory (GST), is used to investigate a large amount of unknown information using a small amount of information in a system containing incomplete data, which was first proposed by Professor Julong Deng in the 1980s [15]. In the grey prediction model GM (n, m), ‘n’ and ‘m’ represent the order of the differential equation and the number of variables, respectively. GM (1,1) model is a classical primary time-series predictive model, representing the first-order model with a single variable. It is most commonly used in grey system theory due to its virtue of “strong adaptability, simple model, easy parameter changes” [16, 17]. Given its unique advantages, GM (1,1) model is widely used to predict in the industries of energy [18], environment [19], medical and health institutions [16, 20]. Therefore, in the present study we used GM (1,1) model to analyze the impact of COVID-19 pandemic on the epidemiological changes of STDs in China. This study was approved by the ethical review committee of Shanghai Skin Disease Hospital (Shanghai, China; approval number: 2022-07) and will be the first research to analyze the situation of STDs during the COVID-19 pandemic in China.

## Methods

### Data collection

HIV infection, syphilis and gonorrhea are national statutory infectious diseases in China, therefor the reported incidence of each STD from 2008 to 2020 was collected from the website of Administration for Disease Control and Prevention of China [21], shown in Additional file 1: Table S1. All data are publicly available.

### Data handling

First, we collected the annual general situation document of national statutory infectious diseases on the official website, which included all reported data of each statutory infectious diseases. Then we screened out the incidence of HIV infection, syphilis and gonorrhea from the documents, respectively, all collected data were dealt with in Excel.

### The establishment of GM (1,1) model

As we know, China revised *the Law of the People’s Republic of China on Prevention and Control of Infectious Diseases* for the second time in 2013 [22], which further strengthened the prevention and control of infectious diseases. Therefore, only data of the actual incidence of each STD from 2013 to 2018 were used to establish GM (1,1) model considering the impact of policies. Then we calculated the predictive incidence of each STD in 2019, 2020 and 2021, which approximately represented the incidence value without influence of COVID-19 pandemic. We further calculated the absolute percentage error (APE) between actual incidence and predictive value in 2019 and 2020. And we then estimated the extent of impact of the COVID-19 pandemic on the epidemiological changes of STDs in China, according to the difference between the APE in 2019 and 2020.

The grey prediction has three basic operations: Cumulative Generation Operator (AGO), Inverse Accumulated Generating Operation (IAGO) and Grey Model (GM) [23]. The steps of establishment and accuracy evaluation metrics of GM (1,1) model are provided in Additional file 1 in detail.

### Statistical analysis

All data were analyzed using SPSS 26.0 software, and the trend Chi-square test was adopted to conduct statistical analysis of the data trends which are described as χ^2^trend. Finally, *p* < 0.05 were considered as statistically significant. The construction, operation and verification of GM (1,1) model was done by using Python software.

## Results

### The epidemiological status of STDs in China

**Fig. 1:**
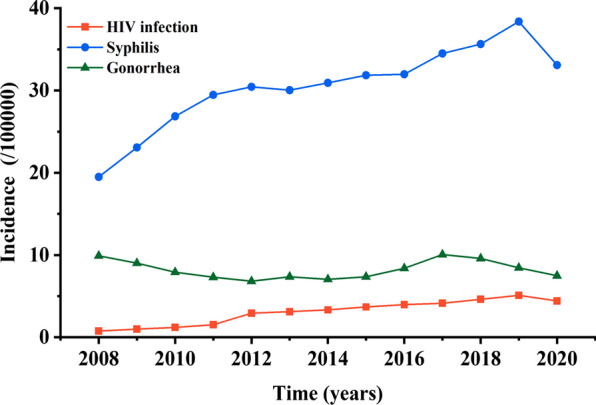
The incidence of each STD from 2008 to 2020 in China

The results showed that the incidence of HIV infection (χ^2^trend = 10.588, *p* = 0.001) and syphilis (χ^2^trend = 9.893, *p* = 0.002) presented an increasing trend from 2008 to 2019, with an annual growth rate of 18.89% and 6.35%, respectively (Fig. 1). While for gonorrhea, it was constantly fluctuating in these years (χ^2^trend = 0.159, *p* = 0.69) (Fig. 1). The incidence of HIV infection, syphilis and gonorrhea declined by 13.1%, 13.8% and 11.4% in 2020 (4.43/100,000, 33.08/100,000, 7.49/100,000), as compared with that in 2019 (5.1/100,000, 38.37/100,000, 8.45/100,000) (Fig. 1), respectively. Therefore, we further used GM (1,1) model to analyze the extent of COVID-19 pandemic impact on STDs in China.

### The established GM (1,1) model of HIV infection, syphilis and gonorrhea

GM (1,1) model was established based on the original data of the incidence of HIV infection, syphilis and gonorrhea from 2013 to 2018, respectively. The fixed values of parameters ‘a’, ‘b’, and the formula of predictive incidence were shown in Table 1.

**Table 1:** The GM (1,1) model of each STD

	a	b	b/a	GM (1,1) model
HIV infection	− 0.077	2.998	− 39.026	${\widehat{x}}^{(0)}(k+1)=3.117{e}^{0.077k}$
Syphilis	− 0.037	28.922	− 785.691	${\widehat{x}}^{(0)}(k+1)=29.482{e}^{0.037k}$
Gonorrhea	− 0.090	6.050	− 67.028	${\widehat{x}}^{(0)}(k+1)=6.420{e}^{0.090k}$

The model test indexes MAPE (mean absolute percentage error), *P*^o^ (the accuracy of GM (1,1) model), *C* (posterior variance ratio), *P* (posterior probability) of HIV infection, syphilis and gonorrhea were shown in Table 2. According to Lewis’ criterion (Additional file 1: Table S2) and the Posterior Deviation criterion (Additional file 1: Table S3), the GM (1,1) model of HIV infection, syphilis and gonorrhea were all graded “Excellent” and “Superior”. This indicated that the established GM (1,1) model can be best used to popularize and predict the incidence of each STD. The predictive value and actual incidence of each STD were shown in Table 2.

**Table 2:** Forecasting results of the GM (1,1) model in each STD

Years	HIV infection			Syphilis			Gonorrhea		
	Actual incidence (1/100,000)	Predictive incidence (1/100,000)	APE (%)	Actual incidence (1/100,000)	Predictive incidence (1/100,000)	APE (%)	Actual incidence (1/100,000)	Predictive incidence (1/100,000)	APE (%)
2013	3.12	–	–	30.04	–	–	7.36	–	–
2014	3.33	3.37	1.20	30.93	30.59	1.10	7.05	7.02	0.43
2015	3.69	3.64	1.36	31.85	31.75	0.31	7.36	7.69	4.48
2016	3.97	3.93	1.01	31.97	32.94	3.03	8.39	8.41	0.24
2017	4.14	4.24	2.42	34.49	34.18	0.90	10.06	9.20	8.55
2018	4.62	4.58	0.87	35.63	35.47	0.45	9.59	10.07	5.01
MAPE (%)	1.37			1.16			3.74		
*P*^o^ (%)	98.63			98.84			96.26		
*C*	0.012			0.052			0.132		
*P*	1			1			1		
2019	5.1	4.95	2.94	38.37	36.81	4.07	8.45	11.02	30.41
2020	4.43	5.34	20.54	33.08	38.19	15.45	7.49	12.05	60.88
2021	–	5.77	–	–	39.64	–	–	13.19	–

### For HIV infection

The predictive incidence of HIV infection in 2019 and 2020 were 4.95/100,000, 5.34/100,000 (Table 2), respectively. The APE between the predictive incidence and actual value in 2020 (20.54%) was about 7 times of that in 2019 (2.94%) (Table 2). The error value between the predictive and actual incidence of HIV infection in 2020 was about 0.91/100,000. The result showed a striking difference between the predictive incidence and the actual value of HIV infection in 2020, indicating that the actual incidence was dramatically reduced during COVID-19 pandemic as compared to that in 2019.

### For syphilis

The predicted incidence of syphilis were 36.81/100,000, 38.19/100,000 in 2019 and 2020 (Table 2), respectively. The value of APE in 2020 (15.45%) was about 4 times of that in 2019 (4.07%), which showed a significant difference between predictive and actual incidence of syphilis in 2020. And the error value was about 5.11/100,000 between the predictive incidence and actual value of syphilis in 2020. The result indicated that COVID-19 may have similar impact to that of HIV infection in 2020 on the actual incidence of syphilis.

## For gonorrhea

As shown in Table 2, the predictive incidence of gonorrhea were 11.02/100,000 and 12.05/100,000 in 2019 and 2020, respectively. The APE in 2020 (60.88%) was about 2 times of that in 2019 (30.41%). And the error value between the predictive and actual incidence was about 4.56/100,000 in 2020. The difference between the predictive incidence and actual value in 2020 is the result of the influence of COVID-19 pandemic.

From the analysis of the incidence of the three STDs during and before COVID-19 pandemic, we found that the epidemiological feature of STDs in China was significantly changed during COVID-19 pandemic and there was a dramatic decrease.

### Forecasting results of each STD in 2021

The incidence of HIV infection, syphilis and gonorrhea would be 5.77/100,000, 39.64/100,000 and 13.19/100,000 in 2021 based on our model (Table 2), respectively. It reflects a trend of continuously increasing incidence of STDs in the following year.

## Discussion

STDs remain the most common contagious diseases worldwide. According to the data of WHO, the estimated new infections of syphilis and gonorrhea were about 6 million and 78 million globally in 2016 [24]. By 2020, about 77.5 million people have been infected with HIV worldwide, and approximately 34.7 million people have died from AIDS-related diseases since the first case of HIV infection was reported [25].

In China, HIV infection, syphilis and gonorrhea are national statutory infectious diseases, and the incidence of these three STDs showed a trend of increase in the recent 20 years [13, 14]. According to the latest reports from the *Administration for Disease Control and Prevention of China* [21], there were 62,167, 464,435 and 105,160 people infected with HIV, syphilis and gonorrhea in 2020, respectively, which were dramatically decreased by 12.7% (71,204), 13.3% (535,819) and 10.8% (117,938) compared with that in 2019. As our results shown (Fig. 1), the incidence of HIV infection and syphilis presented a trend of increase from 2008 to 2019, while the incidence of gonorrhea presented a form of smooth fluctuation. However, this trend of increase was broken in 2020. The incidence of HIV infection, syphilis and gonorrhea dramatically declined by 13.1%, 13.8% and 11.4% in 2020 compared with that in 2019, respectively, as shown in our results (Fig. 1).

China experienced the COVID-19 pandemic at the beginning of 2020. Though the epidemic first broke out in Wuhan, the run on medical resources caused panic across the country [26]. At the same time, the government quickly introduced policies to contain the spread of the epidemic, such as city lockdown, home-staying, keeping social distance, wearing masks, hands hygiene, emergency medical assistance for COVID-19, which effectively prevent and control the spread of COVID-19 to some extent [12, 27, 28]. However, all those measures have had a profound impact on sexual health and behavior of individual [4]. As reported by Bonett, Stephen et al. [29] the disruptions in STDs testing infrastructure during the COVID-19 pandemic threaten to impact STDs service. The STDs test counts decreased and test positivity increased during the pandemic period. According to the report from Greece, Cuba and other countries, the epidemiology of STDs has been widely affected by the COVID-19 pandemic, and presented a trend of dramatic decrease [5, 8–10]. The same situation could happen in China, in our unpublished data at the STD clinic in Shanghai Skin Disease Hospital, we found that patients with STDs interrupted follow-up at the beginning of the COVID-19 outbreak, and the first visit patients sharply declined. Our observed reduction of hospital visits in patients with STDs during COVID-19 pandemic, similar to other non-COVID-19 related disease reported elsewhere [30]. Two out of the three STD clinics of our hospital which were located in the center of Shanghai were closed meanwhile. Based on a survey in our hospital (data unpublished), people reduced their high-risk sexual behavior for fear of being infected with COVID-19. In this study, we analyzed the extent of the impact of COVID-19 pandemic on the epidemiological trend of STDs in China. We found that the APE of HIV infection, syphilis and gonorrhea in 2020 was about 7 times, 4 times and 2 times of that in 2019, which revealed a significant change of the epidemiology of STDs in 2020, especially HIV infection and syphilis. Though it is not clear whether the sharp decline in the incidence of STDs in 2020 was due to the fact that the STDs was hidden by the COVID-19 pandemic, or that STDs had been controlled because of the policy of “city lockdown, home-staying, keeping social distance”, or that people reduced their high-risk sexual behavior during COVID-19 pandemic, due to the fear of and anxiety about the highly contagious characteristics of COVID-19, we suspect that both might contribute to the decline of the incidence of STDs in China.

Although vaccination for the COVID-19 is one of the most effective methods for the general improvement of social safety and individual health [31] and the non-pharmaceutical prevention strategies, such as keeping social distance, using face mask could play a notable role in containing the transmission of COVID-19 for vast majority of the population [32], the strict prevention strategies such as city lockdown, home-staying are still performed to reduce COVID-related morbidity and mortality in China. However, just like what previous researchers pointed out “the intensity of interventions required needs to be balanced against the wider health risks that diverting all attention to a single disease could entail” [7]. Similar to ours, researches of other countries have shown that the COVID-19 epidemic has great impact on STDs [7, 11]. Therefore, the prevention and control of STDs still needs more attention from the society during the COVID-19 epidemic.

In 2020, under the influence of COVID-19, the prevention and treatment of STDs may somewhat be hindered. The research reported by Pascoal, et al. highlighted the role of mental health in the impact of COVID-19 on sexual health [4]. Due to the increasing difficulty in the supervision of STDs during COVID-19, the real situation of the epidemiology of STDs may be underestimated, and STDs incidence might rebound after a long-term development, resulting in a more serious disease burden of society. Therefore, except for emphasizing those well-known policies to prevent STDs [33], such as education on sexual healthy, condom use and partner notification, it is very important that in the event of major pandemics, medical resources should be reasonably allocated, especially for people with HIV, for higher acquisition rates and a poorer outcome of COVID-19 infection in people living with HIV are expected [34]. As such, when managers strengthening pandemic control, the medical aid resources for STDs should be protected from damage, to prevent increasing heavier burden of social diseases in the long run. Different guidelines should be designed and accurate evaluation is required for STDs patients with COVID-19. A better management with special consideration must be given to patients with STDs, such as against discrimination on STDs, encourage self-testing and online medical care, offer necessary remote consultation and medical support, express delivery drugs and special medical pass when lockdown during the COVID-19 pandemic. Evidence has shown that mobile health (mHealth) in mobile apps has been used to enable health care providers to reach out to vulnerable individuals, to provide counselling, health-related education, and treatment [35]. As shown in a study by Pant Pai et al. [36], an unsupervised HIV self-testing strategy using an internet-based mHealth provided counselling and treatment among patients testing positive in South Africa. It’s worth noting that the mobile app organization should boost health care professionals’ core competence with regard to telemedicine, and effectively supervise the quality of mHealth. Moreover, people’s mental health should be guided in a timely manner. The official notice should play a positive role in guiding the real-time reporting of the pandemic, guiding the public to prevent diseases with scientific means, to reduce the fear and anxiety of people caused by the pandemic.

This study also predicted the burden of STDs in 2021, which showed a trend of increasing incidence, warning that we should always keep an eye on the transmission of STDs seriously in China. And with the pandemic well controlled in China for that time, a much little difference between predictive and real infection rate would be strong evidence to prove our findings.

There are some limitations in our study. As we mentioned above, GM (1,1) model is a time-series model [15], and “Simplicity and efficiency” are its advantages. However, using a time-series model to predict the trend of STDs has its inevitable limitations, because the time-series predictive model mainly emphasizes the influence of time factor, which cannot comprehensively show the influence of various other factors. While for STDs, the sexual behavior of the population dictates the incidence and trend of the pandemic. As mentioned, the pandemic of COVID-19 could have impacted on the epidemiology of STDs in several ways. Positively, it could reduce the chance of high-risk sexual behaviors. Because of social-distancing and lockdown, casual and commercial sexual intercourse would decline and the incidence of STDs would be reduced. Negatively, COVID-19 might disrupt healthcare services, such as HIV testing, following and distribution of antiretroviral therapy (ART) drugs, and all these increases the risk of STDs transmission. Furthermore, the number of diagnoses during COVID-19 also drops probably due to less testing has been conducted. Therefore, the GM (1,1) model in this study might not reflect the actual transmission of the three diseases.

### Recommendations

Based on the analysis of the impact of COVID-19 pandemic on STDs, we recommend that vaccination for the COVID-19 should be given to people, including those with STDs. And the non-pharmaceutical prevention strategies must be undertaken by the STDs patients to protect themselves during the pandemic. Medical aid resources for STDs should be reasonably allocated and protected from damage. Special guidelines should be designed for STDs patients with COVID-19, including self-testing, online medical care, remote consultation and medical support, timely diagnosis and treatment guarantee, special medical pass when lockdown during the COVID-19 pandemic.

## Conclusions

COVID-19 pandemic has influenced people’s sexual behavior and health seeking behavior. This research revealed the significant impact of COVID-19 pandemic on the epidemiological trend of STDs in China for the first time. Our findings highlight that people with STDs are encouraged to be vaccinated and must undertake preventive measures to protect themselves during the pandemic. A better management with special consideration must be given to STDs patients with or without COVID-19 and transformative strategies in STDs control is highly needed under the context of the long-term effects of COVID-19 pandemic in the future.

## Supplementary Information

**Additional file 1:** Supplemental methods. **Table S1.** The actual incidence of each STD from 2008 to 2020 in China (1/100000). **Table S2** Lewis’ criterion for model evaluation. **Table S3** The Posterior Deviation criterion of predictive accuracy for the GM (1,1).

## Data Availability

The original data was obtained from the website of Administration for Disease Control and Prevention of China, publicly available at http://www.nhc.gov.cn/jkj/new_index.shtml [21]. All data generated or analysed during this study are included in this published article and its additional information files.

## Abbreviations

COVID-19: Coronavirus disease 2019
STD: Sexually transmitted disease
APE: Absolute percentage error
MAPE: Mean absolute percentage error
*P*^o^: The accuracy of GM (1,1) model
*C*: Posterior variance ratio
*P*: Posterior probability
