# Supplementary material for: Impact of COVID-19 pandemic on the epidemiology of STDs in China: based on the GM (1,1) model
Source: BMC Infect Dis. 2022 Jun 4;22:519. doi: 10.1186/s12879-022-07496-y (PMC9166241; doi:10.1186/s12879-022-07496-y)
Supplement: Supplementary file 1 — Additional file 1: Supplemental methods. Table S1. The actual incidence of each STD from 2008 to 2020 in China (1/100000). Table S2 Lewis’ criterion for model evaluation. Table S3 The Posterior Deviation criterion of predictive accuracy for the GM (1,1). [file 12879_2022_7496_MOESM1_ESM.pdf]

Supplemental Methods

Table S1 The actual incidence of each STD from 2008 to 2020 in China (1/100000)

Years	HIV infection	Syphilis	Gonorrhea
**2008**	0.76	19.49	9.9
**2009**	1	23.07	9.02
**2010**	1.2	26.86	7.91
**2011**	1.53	29.47	7.31
**2012**	2.93	30.44	6.82
**2013**	3.12	30.04	7.36
**2014**	3.33	30.93	7.05
**2015**	3.69	31.85	7.36
**2016**	3.97	31.97	8.39
**2017**	4.14	34.49	10.06
**2018**	4.62	35.63	9.59

The establishment of GM (1,1) model

The steps of establishment of GM (1,1) model are as follows:

Step 1. Suppose the original data sequence $X^{(0)}$ with n sample showed in Eq.(1).

$$X^{(0)} = \left(x^{(0)}(1), x^{(0)}(2), x^{(0)}(3), \cdots, x^{(0)}(n)\right) \qquad n \geq 4 \tag{1}$$

Where $x^{(0)}(i) > 0, i = 1,2,3 \cdots n$. Then the $X^{(0)}$ is transformed to the monotonically increasing series $X^{(1)}$ using a first time AGO (1-AGO) showed in Eq.(2). This aims to weaken the random factors of the original time series data and strengthen its regularity. After that, we establish the differential equation of the generated number from which the data can be predicted and estimated.

$$X^{(1)} = \left(x^{(1)}(1), x^{(1)}(2), x^{(1)}(3), \cdots, x^{(1)}(n)\right) \tag{2}$$

where,

$$x^{(1)}(k) = \sum_{i=1}^{k} x^{(0)}(i) \qquad k = 1,2,3 \cdots, n \tag{3}$$

Step 2. A first-order grey differential equation is formed to obtain GM (1,1) model shown in Eq.(4).

$$x^{(0)}(k) + aZ^{(1)}(k) = b \qquad k = 2,3,4\cdots,n \tag{4}$$

where,

$$Z^{(1)}(k) = \frac{1}{2}\left[x^{(1)}(k) + x^{(1)}(k-1)\right] \qquad k = 2,3,4\cdots,n \tag{5}$$

Where 'a' represent the developing coefficient and 'b' represent the driving coefficient. $Z^{(1)}(k)$ is the background value of $x^{(0)}(k)$.The two parameters 'a'and 'b' can be estimated by the least square method $[a,b]^{\mathrm{T}}$ shown in Eq.(6).

$$\begin{bmatrix} a \\ b \end{bmatrix} = \left[\mathrm{B}^{\mathrm{T}} \quad \mathrm{B}\right]^{-1} \mathrm{B}^{\mathrm{T}}\mathrm{Y} \tag{6}$$

Where 'Y' is the constant vector, and 'B' is the accumulated matrix showed in Eq.(7) and Eq.(8).

$$Y = \begin{bmatrix} x^0(2) \\ x^0(3) \\ x^0(4) \\ \vdots \\ \vdots \\ x^0(n) \end{bmatrix} \tag{7}$$

$$B = \begin{bmatrix} -Z^{(1)}(2) & 1 \\ -Z^{(1)}(3) & 1 \\ -Z^{(1)}(4) & 1 \\ \vdots & \vdots \\ \vdots & \vdots \\ -Z^{(1)}(n) & 1 \end{bmatrix} \tag{8}$$

Step 3. The approximate differential equation (whitening equation) model is established by using the discrete data sequence as Eq.(9).

$$\frac{dX^{(1)}}{dk}+aX^{(1)}=b \tag{9}$$

The predicted value, $\hat{x}^{(1)}(k+1)$, can be obtained by solving the differential equation with initial condition $x^{(1)}(1)=x^{(0)}(1)$.

$$\hat{x}^{(1)}(k+1)=\left[x^{(0)}(1)-\frac{b}{a}\right]e^{-ak}+\frac{b}{a} \qquad k=1,2,3\cdots,n \tag{10}$$

Finally, by means of the IAGO, the predicted value, $\hat{x}^{(0)}(\mathrm{k}+1)$, can be calculated as follows:

$$\hat{x}^{(0)}(k+1)=\hat{x}^{(1)}(k+1)-\hat{x}^{(1)}(k) \qquad k=1,2,3\cdots,n \tag{11}$$

therefore,

$$\hat{x}^{(0)}(k+1)=(1-e^{a})\left(x^{(0)}(1)-\frac{b}{a}\right)e^{-ak} \qquad k=1,2,3\cdots,n \tag{12}$$

Note that $\hat{x}^{(1)}(1)=\hat{x}^{(0)}(1)$ holds.

The accuracy evaluation metrics of GM (1, 1) Model

Prediction accuracy is an important criterion for evaluating the performance of a forecasting model. In this study, two ways were used to estimate the accuracy of the GM (1, 1) model: the absolute error size test and the posterior deviation test [1].

(1) *The Absolute Error Size Test*. It is used to observe whether the absolute error meets the requirements by comparing the predicted incidence and the actual value of STDs in this study. The APE is showed as Eq.(13).

$$\mathrm{APE}=\left|\frac{x^{(0)}(i)-\hat{x}^{(0)}(i)}{x^{(0)}(i)}\right|\times 100\% \tag{13}$$

The mean absolute percentage error (MAPE) of GM (1,1) is used to measure the prediction performance with respect to $x^{(0)}(k)$ ($k = 2,3,4\cdots,n$)can be calculated as Eq.(14).

$$\text{MAPE}=\frac{1}{n}\left(\sum_{i=2}^{n}\left|\frac{x^{(0)}(i)-\hat{x}^{(0)}(i)}{x^{(0)}(i)}\right|\right)\times 100\% \tag{14}$$

Meanwhile, Lewis' criterion shown in Table S2 is used to illustrate the prediction ability of the model [2]. According to that the established GM (1.1) model can be graded four levels "Excellent, Good, Reasonable and Incorrect", respectively. The better the grading, the better the predictive effectiveness of the model.

Table S2 Lewis' criterion for model evaluation [2] [a]

MAPE（%）	Prediction performance
＜10	Excellent
10-20	Good
20-50	Reasonable
＞50	Incorrect

[a] Reproduced with permission from Ref. [2].Copyright 2021 Elsevier.

The accuracy of GM (1, 1) model is P^{o} showed as Eq.(15).

$$P^{o} = (1-\text{MAPE})\times 100\% \tag{15}$$

General requirement is $P^{o} > 80\%$.

(2) *The Posterior Deviation Test*. It is tested according to the probability distribution of residual. The posterior variance ratio *C and* the posterior probability *P* are two main indicators used to evaluate the accuracy of the model.

Calculate the a posteriori variance ratio *C* as Eq.(16).

$$C = \frac{S_2}{S_1} \tag{16}$$

where,

$$S_1 = \sqrt{\frac{1}{n}\sum_{k=1}^{n}\left[x^{(0)}(k) - \bar{x}\right]^2} \tag{17}$$

and,

$$S_2 = \sqrt{\frac{1}{n}\sum_{k=1}^{n}\left[\varepsilon(k) - \bar{\varepsilon}\right]^2} \tag{18}$$

Calculate the posterior probability P as Eq.(19).

$$P = P\left\{\left|\varepsilon(k) - \bar{\varepsilon}\right| < 0.6745 S_1\right\} \tag{19}$$

where,

$$\bar{x} = \frac{1}{n}\sum_{k=1}^{n} x^{(0)}(k) \tag{20}$$

$$\bar{\varepsilon} = \frac{1}{n}\sum_{k=1}^{n} \varepsilon(k) \tag{21}$$

$$\varepsilon(k) = x(k) - \hat{x}(k) \tag{22}$$

The smaller the C value, the more concentrated the difference between the model predicted value and the actual value. Meanwhile, the larger the P value, the less difference between the residual and the residual mean, and the higher fitting accuracy of the GM (1,1) model. According to the size of the value of C and P, the predictive accuracy rating of the established GM (1,1) model can be graded into four levels “Superior, Qualified, Marginal and Disqualified” [3], as shown in Table S3.

Table S3 The Posterior Deviation criterion of predictive accuracy for the GM (1,1) [3][b]

Predictive accuracy class	posterior variance ratio (*C*)	posterior probability (*P*)
**Superior**	⩽0.35	⩾0.95
**Qualified**	0.35＜*C*⩽0.50	0.80⩽*P*＜0.95
**Marginal**	0.50＜*C*⩽0.65	0.70⩽*P*＜0.80
**Disqualified**	＞0.65	＜0.70

[b] Reproduced with permission from Ref. [3]. Copyright 2019 Medical science monitor (CC BY-NC-ND 4.0).

References

1. Wang Y, Wei F, Sun C, Li Q. The Research of Improved Grey GM (1, 1) Model to Predict the Postprandial Glucose in Type 2 Diabetes. Biomed Res Int. 2016;2016:6837052.
2. Luo X, Duan H, Xu K. A novel grey model based on traditional Richards model and its application in COVID-19. Chaos Solitons Fractals. 2021;142:110480.
3. Ye X, Liu J, Yi Z. Trends in the Epidemiology of Sexually Transmitted Disease, Acquired Immune Deficiency Syndrome (AIDS), Gonorrhea, and Syphilis, in the 31 Provinces of Mainland China. Medical science monitor : international medical journal of experimental and clinical research. 2019;25:5657-65. https://doi.org/10.12659/msm.915732.
